# Estimation of the solubility parameters of model plant surfaces and agrochemicals: a valuable tool for understanding plant surface interactions

**DOI:** 10.1186/1742-4682-9-45

**Published:** 2012-11-14

**Authors:** Mohamed Khayet, Victoria Fernández

**Affiliations:** 1 Department of Applied Physics I, Faculty of Physics, Universidad Complutense, Avda Complutense s/n, 28040, Madrid, Spain; 2 Genetics and Eco-physiology Research Group, School of Forest Engineering, Technical University of Madrid, Ciudad Universitaria s/n, 28040, Madrid, Spain

**Keywords:** Agrochemicals, Cuticle, Plant surfaces, Solubility parameter, Waxes

## Abstract

**Background:**

Most aerial plant parts are covered with a hydrophobic lipid-rich cuticle, which is the interface between the plant organs and the surrounding environment. Plant surfaces may have a high degree of hydrophobicity because of the combined effects of surface chemistry and roughness. The physical and chemical complexity of the plant cuticle limits the development of models that explain its internal structure and interactions with surface-applied agrochemicals. In this article we introduce a thermodynamic method for estimating the solubilities of model plant surface constituents and relating them to the effects of agrochemicals.

**Results:**

Following the van Krevelen and Hoftyzer method, we calculated the solubility parameters of three model plant species and eight compounds that differ in hydrophobicity and polarity. In addition, intact tissues were examined by scanning electron microscopy and the surface free energy, polarity, solubility parameter and work of adhesion of each were calculated from contact angle measurements of three liquids with different polarities. By comparing the affinities between plant surface constituents and agrochemicals derived from (a) theoretical calculations and (b) contact angle measurements we were able to distinguish the physical effect of surface roughness from the effect of the chemical nature of the epicuticular waxes. A solubility parameter model for plant surfaces is proposed on the basis of an increasing gradient from the cuticular surface towards the underlying cell wall.

**Conclusions:**

The procedure enabled us to predict the interactions among agrochemicals, plant surfaces, and cuticular and cell wall components, and promises to be a useful tool for improving our understanding of biological surface interactions.

## Background

Plant surfaces play a major role in protection against multiple potential biotic and abiotic stress factors [1]. To adapt to these multiple functions, the plant epidermis has developed various characteristics, including specialised cell types such as trichomes or stomata [2]. Epidermal cells are surrounded by a cell wall, which plays a crucial structural and physiological role in plant development and survival [3].

Differentiation and maintenance of the epidermis are essential for plant growth and survival and require continuous cross-talk between epidermal cells and their immediate environment [2]. Epidermal cells also provide mechanical support by adhering strongly to each other via a strengthened cell wall, which is usually noticeably thicker on the external surface. In addition to the asymmetrical deposition of cell wall material, epidermal cells secrete a lipid-rich cuticle specifically into the thickened external cell wall matrix [2]. Therefore, the cuticle may be considered a cutinised cell wall, emphasizing the heterogeneous nature of this layer and its interconnection with the cell wall beneath [4]. The main protective role of the cuticle is related to the prevention of uncontrolled exchange of water and gases between the plant and the surrounding environment [5]. The functional relevance of the cuticle to plant growth and survival is evidenced by the significant commitment of epidermal cells to cuticle production [6].

The cuticle is made of a bio-polymer matrix, waxes that are deposited on to (epicuticular) or intruded into (intracuticular) this matrix, and variable amounts of polysaccharides and phenolics [4,7]. It is an asymmetric membrane [8] generally comprising three distinct layers from the outer to the inner side of the organ, namely: (i) the epicuticular wax layer, (ii) the “cuticle proper” containing waxes and cutin and/or cutan, and (iii) the “cuticular layer” composed of cutin and/or cutan and a high polysaccharide content [9].

Waxes commonly constitute 20 to 60% of the cuticle mass and are complex mixtures of straight chain aliphatics [6]. Wax composition and structure can vary among different species, organs, states of development, and environmental and stress conditions during growth [10,11]. The mechanisms of epicuticular wax formation and regeneration have been assessed in some studies [12] and it has been proposed that cuticular transpiration is the driving force behind wax movement through the cuticle [13,14].

The cuticle matrix is commonly made of cutin, which is a biopolymer formed by a network of inter-esterified, hydroxyl- and hydroxy-epoxy C_16_ and/or C_18_ fatty acids [15]. At least six different types of cuticular ultrastructures have been identified by transmission electron microscopy (TEM) [9], but their relationship to cutin monomer composition remains unclear [7,16]. The formation of cutinsomes, which are spherical nanoparticles resulting from the self-assembly of cutin hydroxyacid monomers in a polar environment, has been demonstrated; cutinsomes have been proposed as building units of the bio-polyester cutin [17].

While cutin is depolymerised and solubilised upon saponification, cuticles from some species contain a non-saponifiable and non-extractable polymer known as cutan, which yields a characteristic series of long chain *n*-alkenes and *n*-alkanes upon flash pyrolysis [18]. Cutin has been found to be the only polymer present in the cuticles of many fruits and leaves of *Solanaceae* and *Citrus* species [9], while different proportions of cutin and cutan have been determined in cuticular membranes extracted from leaves [18] and fruits such as peppers, apples or peaches [19,20].

Major differences in surface topography have been observed in different species and organs, but three hierarchical levels of structuring may occur in association with: (i) the general shape of epidermal cells, (ii) cuticular folds, and (iii) epicuticular wax crystals [21]. For example, the presence of papillae [22] or trichomes [20] can have a major effect on surface topography and wettability at the microscale level. Also, increased surface roughness and surface hydrophobicity have been reported owing to the occurrence of nano-scale structures provided by epicuticular wax crystals [22,23].

Different degrees of wettability of leaves from various species have been reported by measuring water contact angles (e.g., [21,24-26]). In addition, phyllosphere-related factors such as the deposition of aerosols or microorganisms can lead to plant surface heterogeneity [27,28], especially in urban or polluted habitats [29]. However, non-wettable surfaces have been observed to accumulate particles more slowly than wettable ones [30].

Recently Fernández et al. [20] estimated the surface free energy, polarity and work of adhesion of a model pubescent surface and proposed the implementation of membrane science approaches to exploring the physical-chemical properties of plant surfaces. It has been suggested that the cuticle acts as a “solution-diffusion” membrane for the diffusion of some solvents and solutes [31,32]. To analyse the permeability of the plant cuticle to solutes and solvents, both the solubility and diffusivity of the compounds must be taken into consideration. While diffusivity is a kinetic parameter associated with the molecular size of a compound in relation to the structure of the matrix, solubility is a thermodynamic parameter that indicates the affinity of a given chemical for the cuticle. Therefore, and as a preliminary step towards the evaluation of plant cuticle permeability, we have analysed for the first time the solubility of model plant surfaces and chemical constituents in relation to agrochemicals of commercial significance, following a thermodynamic approach. Prediction of solubility parameters is commonly used, for example, in the design and fabrication of polymeric membranes [33,34], in the coating industry [35] and also in pharmacology [36]. However, with the exception of the human skin [37,38], this procedure has not so far been applied to estimating the properties of biological surfaces.

As model plant surfaces, peach and pepper fruits were selected since they contain alkanes as major wax constituents but have significantly different surface topographies. Juvenile *Eucalyptus globulus* leaves, which are covered with a dense layer of nano-tubes and contain *β*-diketones as dominant waxes, were also evaluated for comparison.

For model plant surfaces, cuticular constituents and agrochemicals, the following hypotheses were tested: (i) is it possible to predict the solubility of plant surface constituents and the affinity of agrochemicals for plant surfaces? and (ii) can solubility parameters be used to estimate the properties of the plant cuticle?

## Materials and methods

### Plant material

The plant materials analysed correspond to intact, undamaged mature peaches (*Prunus persica* (L.) Batsch. cv. ‘Calrico’), red bell peppers (*Capsicum annum* L. cv. ‘Genil’) and juvenile *Eucalyptus* leaves (*Eucalyptus globulus* Labill. ssp. *globulus*).

### Epicuticular waxes, cutin monomers and cell wall polysaccharides

**Figure 1:**
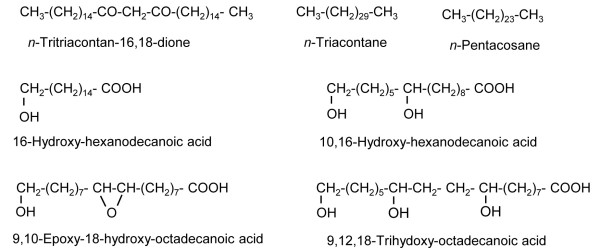
Molecular structures of the cuticular constituents evaluated.

The properties of the major wax constituents present in *Eucalyptus* leaves, bell peppers and peach fruits were used for calculating the solubility parameters (Figure 1, Table 1). Alkanes are the dominant class of compounds covering the surface of the peach fruits analysed [20]. Alkanes are also the dominant class of wax compounds extracted from pepper fruits, followed by triterpenoids such as *α-* or *β*-amyrin [39-41]. *Beta*-diketones are the dominant class of wax compounds in juvenile *Eucalyptus* leaves, but *n*-nonacosane, heptadecan-2-one and *n*-hexacosanal are also present in significant concentrations [42-46].

**Table 1:** **Chemical formula and molar volume of the dominant epicuticular waxes extracted from *Eucalyptus *** **leaves, pepper and peach fruits and of common cutin monomers found in plant cuticles**

**Compound**	**Chemical formula**	**ChemSpider ID**	**Molar volume (cm**^**3**^ **mol**^**-1**^**)**
Epicuticular waxes			
*n*-Hentriacontan-14, 16-dione	C_31_H_60_O_2_	390212	534.4
*n*-Tritriacontan-16, 18-dione	C_33_H_64_O_2_	136445	567.4
*n*-Pentatriacontan-16, 18-dione	C_35_H_68_O_2_	104279	600.5
Heptadecan-2-one	C_17_H_34_O	17031	306.3
Hexadecanal	C_16_H_32_O	956	290.0
*n*-Tricosane	C_23_H_48_	12017	408.1
*n*-Tetracosane	C_24_H_50_	12072	426.7
*n*-Pentacosane	C_25_H_52_	11900	441.2
*n*-Hexacosane	C_26_H_54_	11901	457.1
*n*-Heptacosane	C_27_H_56_	11146	474.2
*n*-Nonacosane	C_29_H_60_	11903	507.2
*n*-Hentriacontane	C_31_H_64_	11904	540.2
*α*-Amyrin	C_30_H_50_O	65921	420.8
Cutin monomers			
16-Hydroxy-hexanodecanoic acid	C_16_H_32_O_3_	10034	284.8
10,16-Hydroxy-hexanodecanoic acid	C_16_H_32_O_4_	390182	282.7
9,10-Epoxy-18-hydroxy-octadecanoic acid	C_18_H_34_O_4_	7994062	309.6
9,12,18-Trihydoxy-octadecanoic acid	C_18_H_36_O_5_	4446065	313.6
Cell wall polysaccharide monomers			
D Glucose	C_6_H_12_O_6_	96749	115.7
D-Galacturonic acid	C_6_H_10_O_7_	76444	109.9

To estimate the solubility parameter range of the cuticle matrix, calculations were carried out with model cutin monomers, which have commonly been identified in plant cuticle monomer analyses [47,48]. The selected *ω*-hydroxy-fatty acids are: 16-hydroxy-hexanodecanoic acid, 10,16-dihydroxy-hexanodecanoic acid, 9,10-epoxy-18-hydroxy-octadecanoic acid, and 9,10,18-trihydoxy-octadecanoic acid (Table 1). Maximal and minimal solubility parameter values were estimated per monomer according to the potential formation of ester bonds.

The solubility parameter of cellulose, a biopolymer formed from unbranched, unsubstituted (1,4)-*β*-D-glucan chains [3], was evaluated by estimating the properties of the D-glucose monomer. The solubility parameter range of pectins was assessed by analysing the structure of homogalacturonans based on *α*-1-4 linked, D-galacturonic acid Table 2; [3,49].

**Table 2:** Characteristics of the chemicals used for estimation of solubility parameters

**Compound**	**Chemical formula**	**Molar volume (cm**^**3**^ **mol**^**-1**^**)**	**ChemSpider ID**	**Activity**
Urea	CH_4_N_2_O	45.2	1143	Fertiliser
Sorbitol	C_6_H_14_O_6_	113.9	5576	Adjuvant
Flutolanil	C_17_H_16_F_3_NO_2_	224.9	43579	Fungicide
Fenoxycarb	C_17_H_19_NO_4_	244.0	46739	Insecticide
Chlorothalonil	C_8_Cl_4_N_2_	152.8	13861400	Fungicide
Formetanate	C_11_H_15_N_3_O_2_	187.5	28856	Insecticide
Esfenvalerate	C_25_H_22_ClNO_3_	341.4	8517510	Insecticide
α-Cypermethrin	C_22_H_19_C_l2_NO_3_	313.0	2809	Insecticide
Triton X-100	C_34_H_62_O_11_	604.5	5388	Surfactant
Brij 35	C_58_H_118_O_24_	1130.6	2006408	Surfactant
Genapol X-80	C_29_H_59_O_9_	552.3	-	Surfactant

### Chemicals

**Figure 2:**
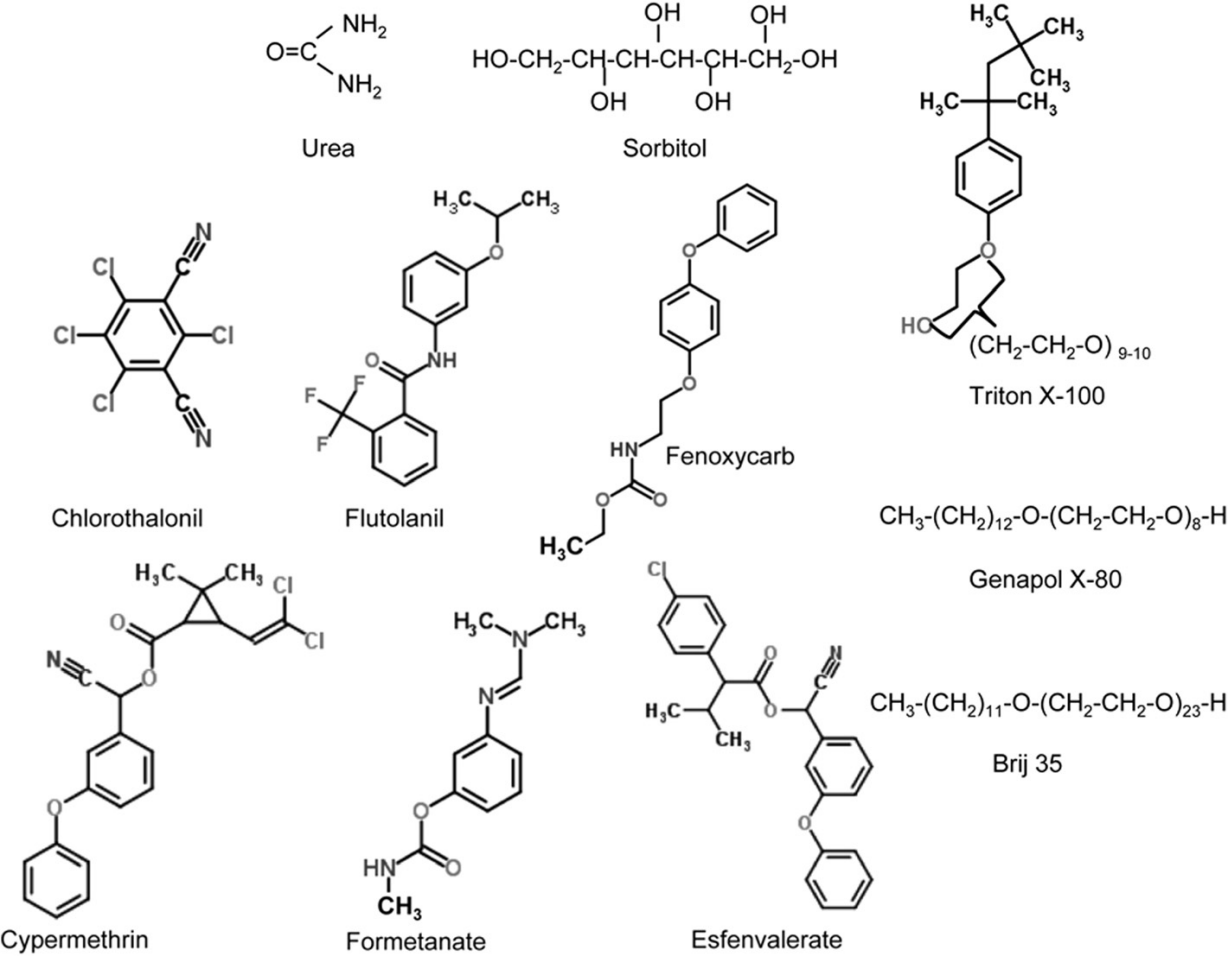
Molecular structures of the agrochemicals selected for calculation of solubility parameters.

Several compounds with different properties and degrees of complexity were selected for calculation of solubility parameters (Figure 2, Table 2). The densities of urea and sorbitol were obtained from the PubChem Bioassay Database (http://pubchem.ncbi.nlm.nih.gov; identification codes: 1176, 5780 and 311, respectively). The molecular structures of plant protection active ingredients (flutolanil, fenoxycarb, chlorothalonil, formetanate, esfenvalerate and cypermethrin) were obtained from ChemSpider with some modifications (Royal Society of Chemistry, UK). Details of the densities of flutolanil, fenoxycarb and fometanate were collected from the Pesticide Properties Database (University of Hertforshire, UK). The densities of chlorothalonil, esfenvalerate and cypermethirn were derived from the European Union Pesticides Database, while data on Genapol X-80 (8 ethylene oxide (EO) units, 13.4 hydrophile-to-lipophile balance (HLB)) and Triton X-100 (assuming 10 EO units and 14.1 HLB) were obtained from Sigma-Aldrich product data-sheets. The density of Brij 35 (23 EO units, 17.1 HLB) was obtained from ChemSpider. Molecular weights were calculated from the number of atoms, and molar volumes were estimated by dividing the molecular weight by the density (Table 2).

### Microscopy

Gold-sputtered intact *Eucalyptus* adaxial leaves, peach and pepper fruit surfaces were examined with a Hitachi S-3400 N (Tokyo, Japan) and a Philips XL30 (Eindhoven, The Netherlands) scanning electron microscope (SEM).

For TEM observations of *Eucalyptus* leaf tissue, approximately 1 mm^2^ sections were cut with a scalpel and fixed in 2% paraformaldehyde plus 2% glutaraldehyde (both from Electron Microscopy Sciences (EMS), Hatfield, USA) for 6 h in ice-cold phosphate buffer (pH 7.2). Samples were subsequently washed five times in phosphate buffer, kept at 4°C overnight, and post-fixed with 1% osmium tetraoxide (TAAB Laboratories, Berkshire, UK) and 1.5% potassium ferrocyanide (Sigma-Aldrich, Munich, Germany) in distilled water (2 h). They were then rinsed in distilled water (3 × 10 min) and dehydrated in an acetone:water (v/v) series of 50, 60, 70, 80, 90, 95 (2 × 10 min each) and 100% (3 × 10 min). The tissues were successively immersed in 1:3 (2 h), 1:1 (2 h) and 1:3 (3 h) Spurr’s resin:acetone (v/v) solutions and kept overnight in pure Spurr’s resin (TAAB Laboratories). The samples were finally placed in moulds and were incubated at 60°C for three days. Ultrathin sections were stained with uranyl acetate (20 min) and lead citrate (4 min; both chemicals from EMS) and were examined by TEM (Jeol JEM-1010, Tokyo, Japan).

### Contact angle measurements and prediction of solubility parameters

Advancing contact angles of drops of double-distilled water, glycerol and diiodomethane (both 99% purity, Sigma-Aldrich) were measured at 25°C using a CAM 200 contact angle meter (KSV Instruments Ltd., Helsinki, Finland). Contact angles were measured on intact *Eucalyptus* adaxial leaf, peach and pepper fruit surfaces (30 repetitions). The plant surfaces analysed were collected from fruits and leaves previously observed by SEM. No materials that could affect contact angle measurements (e.g., salt deposits or microorganisms) were found to be deposited on them.

Two μL drops of each liquid were deposited on to the plant surfaces with a manual dosing system holding a 3 mL syringe (0.5 mm diameter needle). Side view images of the drops were captured at a rate of 10 frames s^-1^. Contact angles were automatically calculated by fitting the captured drop shape to the one calculated from the Young–Laplace equation.

For the three plant surfaces evaluated, the total surface free energy, including its three components (i.e. the Lifshitz-van der Waals (LW), acid (+) and base (−) components), was calculated in addition to the surface polarity and work of adhesion [20].

### Prediction of solubility parameters

The solubility parameter of each plant surface analysed, *δ*_*θ*_, was calculated from the following equation [50]:

(1) δθ=ec12

where *e*_*c*_ (MJ m^-3^) is the cohesive energy density, which is related to the surface free energy, *γ*_*s*_, (mJ m^-2^) as follows:

(2) ec=γs0.7523

The solubility parameter of a material can be calculated from either the cohesive energy (Eqn. 1) or the molar attraction constant, *F* ((MJ/m^3^)^1/2^ mol^-1^), as:

(3) $$\delta =\frac{F}{\upsilon }$$

where *v* is the molar volume (cm^3^ mol^-1^) of the molecule [51].

The solubility parameter has three components taking into account the interactions due to dispersion forces (*δ*_*d*_), polar forces (*δ*_*p*_) and hydrogen (H)-bonding (*δ*_*h*_), and it is expressed as:

(4) $$\delta =\sqrt{\delta_{d}^{2}+\delta_{p}^{2}+\delta_{h}^{2}}$$

According to van Krevelen and Hoftyzer [52], the solubility parameter components can be predicted from group contributions, using the following equations:

(5) $$\delta_{d}=\frac{\sum F_{\mathrm{di}}}{v}$$

(6) $$\delta_{p}=\frac{\sqrt{\sum F_{\mathrm{pi}}^{2}}}{v}$$

(7) $$\delta_{h}=\sqrt{\frac{\sum E_{\mathrm{hi}}}{v}}$$

where *F*_*di*_ and *F*_*pi*_ are the molar attraction constants of the dispersion and polar components, respectively, *E*_*hi*_ is the H-bonding energy and *v* is the molar volume.

The contributions of the functional groups present in the chemicals and plant structural compounds analysed to the solubility parameter components are shown in Table 3. From the solubility parameter components, the total solubility parameter (*δ*) can be calculated from Equation 4, and is hereafter named *δ*_*m*_ for agrochemicals, *δ*_*wax*_ for epicuticular waxes and *δ*_*nm*_ for cutin and polysaccharide monomers.

**Table 3:** **Contributions of structural groups present in the selected molecules to the solubility parameter component**[52]

**Structural group**	***F***_***di ***_**((MJ/m**^**3**^**)** ^**1/2**^ **mol**^**-1**^**)**	***F***_***pi ***_**((MJ/m**^**3**^**)** ^**1/2**^ **mol**^**-1**^**)**	***E***_***hi ***_**(J/mol)**
-CH_3_	420	0	0
-CH_2_-	270	0	0
>CH-	80	0	0
=C<	70	0	0
=CH-	200	0	0
>C<	−70	0	0
	1430	110	0
	1270	110	0
-F	220	0	0
-Cl	450	550	400
-OH	210	500	20000
-O-	100	400	3000
-CO-	290	770	2000
-COO-	390	490	7000
-COOH	530	420	10000
-COH	470	800	4500
-CN	430	1100	2500
>N-	20	800	5000
-NH_2_	280	0	8400
-NH-	160	210	3100
1 plane of symmetry	—	0.50 ×	—
2 planes of symmetry	—	0.25 ×	—
More planes of symmetry	—	0 ×	0 ×

Finally, to evaluate the affinity of a polymer for a solvent [51] or the affinity of an agrochemical for a given plant surface, the following equation was used:

(8) $$\Delta \delta_{wax=}\sqrt{{\left(\delta_{m}-\delta_{\mathrm{wax}}\right)}^{2}}$$

Moreover, to study the affinities of the agrochemicals for plant surfaces as derived from the solubility parameter calculated from contact angle measurements (*δ*_*θ*_), the following equation was applied:

(9) $$\Delta \delta_{\theta =}\sqrt{{\left(\delta_{m}-\delta_{\theta }\right)}^{2}}$$

The results from Equations 8 and 9 imply that the lower the values of Δδ_wax_ and Δδ_θ_, the higher the affinity between agrochemical and plant surface.

## Results

### Surface topography and hydrophobicity

**Figure 3:**
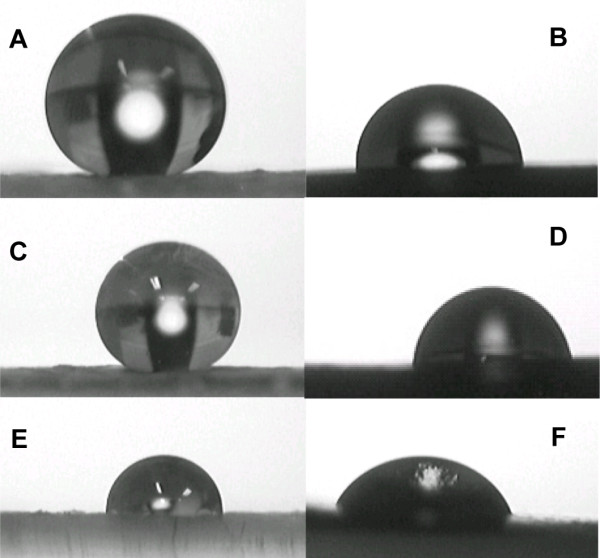
**Contact angle measurements on intact, adaxial *Eucalyptus *(A,C,E) leaf surfaces and pepper fruit surfaces (B,D,F).** Drops of: (**A**,**B**) water, (**C**,**D**) glycerol, and (**E**,**F**) diiodomethane.

The contact angles (in °) of water, glycerol and diiodomethane with plant surfaces are (mean ± standard deviation): 142.6 ± 6.7, 136.5 ± 11.2 and 84.0 ± 7.0 for *Eucalyptus* leaves; 83.4 ± 4.7, 68.6 ± 9.2 and 60.8 ± 6.2 for pepper fruits; and 134.2 ± 7.0, 130.9 ± 7.0 and 55.7 ± 3.9 for peach fruits (see Figure 3 as an illustration of the measurements).

**Figure 4:**
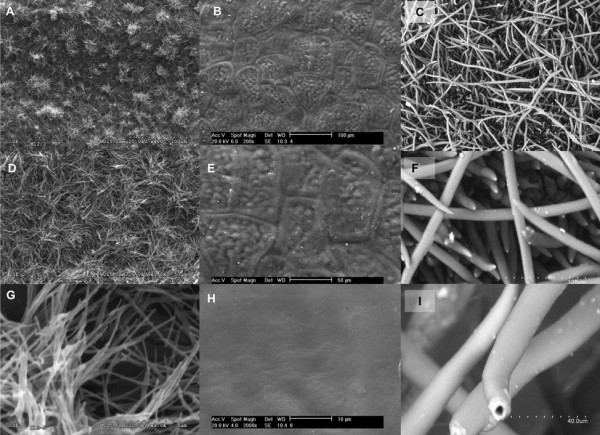
**Scanning electron micrographs of intact plant surfaces.***Eucalyptus* adaxial leaf surfaces: (**A**) ×400, (**D**) ×1,000, and (**G**) ×9,000. Pepper fruit surfaces: (**B**) ×200, (**E**) ×400, and (**H**) ×1,000. Peach fruit surfaces: (**C**) ×100, (**F**) ×500, and (**I**) ×1,300.

The topographies of the plant materials analysed are shown in Figure 4. The adaxial surface of juvenile *Eucalyptus* leaves is densely covered with a network of wax nano-tubes, which can be clearly identified as such at higher magnifications (Figure 4D and G). In contrast, the pepper fruit surface is covered with a pattern of epidermal cells (Figure 4B and E) and epicuticular waxes with no clear structure, yielding a smooth and rather flat surface. The peach surface is densely covered with conspicuous trichomes (approximately 1 mm long and 20 μm thick), which provide a high degree of micro-scale roughness (Figure 4C, F and I) in contrast to the nano-scale surface roughness of *Eucalyptus* leaves (Figure 4G). Given the dense and thick network of micro- (trichomes) and nano- (epicuticular waxes) tubes covering peach fruits and *Eucalyptus* leaves, respectively, we could not determine their roughness by atomic force microscopy.

Adaxial *Eucalyptus* leaf surfaces are almost super-hydrophobic and the peach fruit surface is also very hydrophobic, while the pepper fruit surface is more wettable.

### Surface free energy, polarity, work of adhesion and solubility parameter

Measurement of contact angles with water, glycerol and diiodomethane enabled several plant surface properties to be calculated (Table 4). The total surface free energies of peach and pepper fruits are similar and significantly higher than that measured for *Eucalyptus* leaves (approximately 32.2 versus 17.4 mJ m^-2^, respectively). In all cases, there is a major contribution of the Lifshitz-van der Waals component, while the acid–base component is more significant in pepper fruits. Peach and *Eucalyptus* surfaces have higher contributions from electron acceptor interactions, whilst electron donor interactions predominate in pepper. The lowest and the highest surface polarities correspond to peach and pepper fruits, respectively. The work of adhesion for water and glycerol is much higher in pepper fruits (81.2 mJ m^-2^) than peach fruits and *Eucalyptus* leaves (between 15.0 and 22.1 mJ m^-2^; Table 4). However, the work of adhesion for diiodomethane lies within a similar range to that for peach and pepper fruits, and is significantly lower in *Eucalyptus* leaves.

**Table 4:** **Surface free energy per unit area, Lifshitz van der Waals component *(γ***^***LW***^**), acid–base component (*γ***^***AB***^**) with the contributions of electron donor (γ**^**-**^**) and electron acceptor *(γ***^***+***^**) interactions, total surface free energy ( *γ *), surface polarity *(γ***^***AB***^ ***γ ***^***-1***^**), solubility parameter (δ**_**θ**_**) and work of adhesion (for water, *W***_***w***_**; glycerol, *W***_***g***_**; diiodomethane, *W***_***d***_**) of adaxial *Eucalyptus *** **leaf, pepper and peach fruit surfaces**

	**Surface free energy and its components** **(mJ m**^**-2**^**)**							**Work of adhesion (mJ m**^**-2**^**)**		
Sample	*γ*^*LW*^	*γ*^*-*^	*γ*^*+*^	*γ*^*AB*^	*γ*	*γ*^*AB*^*γ*^*-*1^ (%)	*δ*_*θ*_*(*MJ^1/2^ m^-3/2^)	*W*_*a.w*_	*W*_*a.g*_	*W*_*a.d*_
*Eucalyptus*	15.5	0.2	6.5	1.0	17.4	11.2	10.6	15.0	17.6	56.1
Pepper	28.1	3.9	1.4	4.6	32.7	14.1	17.0	81.2	87.4	75.6
Peach	31.1	0.04	10.0	1.2	32.2	3.7	16.8	22.1	22.1	79.4

Concerning the solubility parameters (*δ*_*θ*_) of the three plant materials analysed (Table 4), *Eucalyptus* leaves exhibit a significantly lower value (10.6 MJ^1/2^ m^-3/2^) than pepper and peach fruit surfaces (approximately 17 MJ^1/2^ m^-3/2^).

### Solubility parameter of epicuticular waxes

The solubility parameters of the most abundant epicuticular waxes (*δ*_*wax*_) of *Eucalyptus* leaves, pepper and peach fruits are shown in Table 5.

**Table 5:** **Total solubility parameter (*δ***_***wax***_**) and solubility parameter components of the most abundant epicuticular waxes of *Eucalyptus *** **leaves, pepper and peach fruits**

	**Solubility parameter components (MJ**^**1/2 **^**m**^**-3/2**^**)**			
Compound	*δ*_*d*_	*δ*_*p*_	*δ*_*h*_	*δ*_*wax*_ (MJ^1/2^ m^-3/2^)
*Eucalyptus* leaf				
*n*-Tritriacontan-16, 18-dione	16.3	1.4	2.7	16.6
*n*-Pentatriacontan-16, 18-dione	16.3	1.3	2.6	16.6
*n*-Hentriacontan-14, 16-dione	16.3	1.4	2.7	16.6
*n*-Nonacosane	16.0	0	0	16.0
Hexacosanal	16.2	1.8	3.1	16.0
Heptadecan-2-one	16.0	2.5	2.6	16.4
Pepper fruit				
*n*-Hentriacontane	16.1	0	0	16.1
*n*-Nonacosane	16.0	0	0	16.0
*n*-Heptacosane	16.0	0	0	16.0
*α,β-*Amyrin	15.2	2.5	2.6	16.7
Peach fruit				
*n*-Pentacosane	16.0	0	0	16.0
*n*-Heptacosane	16.0	0	0	16.0
*n*-Tricosane	16.0	0	0	16.0
*n*-Nonacosane	16.0	0	0	16.0
*n*-Hexacosane	16.0	0	0	16.0
*n*-Tetracosane	16.0	0	0	16.0

The dominant class of compounds in both pepper and peach fruit waxes is *n*-alkanes, which have a *δ*_*wax*_ around 16 MJ^1/2^ m^-3/2^ for the most abundant compounds reported (C_23_ to C_31_*n*-alkanes). However, it is remarkable that such compounds lack polar (*δ*_*p*_) and H-bonding (*δ*_*h*_) solubility parameter components. While *n*-alkanes are the most abundant waxes in peach, a relatively abundant class of triterpenoids (e.g., *α,β*-amyrin) can be found in pepper fruits. Although the presence of such waxes will not significantly modify *δ*_*wax*_ (16.7 MJ^1/2^ m^-3/2^, for *α*,*β*-amyrin), they could potentially facilitate interactions due to polar forces and H-bonding owing to the presence of a functional alcohol group.

With regard to *Eucalyptus* leaves, the dominant class of compounds is *β*-diketones, which have a *δ*_*wax*_ of approximately 16.6 MJ^1/2^ m^-3/2^ and non-zero *δ*_*p*_ and *δ*_*h*_ components owing to the presence of ketone functional groups. Other wax classes often found in *Eucalyptus* leaves are *n*-alkanes (chiefly *n*-nonacosane), alkanals (aldehydes) such as hexacosanal, and ketones (e.g., heptadecan-2-one). These kinds of waxes and some others not included in Table 2 (data not shown) were found to have *δ*_*wax*_ values ranging between 16 and 17 MJ^1/2^ m^-3/2^, but in contrast to *n*-alkanes they all have contributions from *δ*_*p*_ and *δ*_*h*_ because of the presence of aldehyde, ketone and/or alcohol functional groups.

For peach and pepper fruits, the values of *δ*_*θ*_ and *δ*_*wax*_ are within the same range (16 to 17 MJ^1/2^ m^-3/2^). However, a significantly lower *δ*_*θ*_ value (10.6 MJ^1/2^ m^-3/2^*)* was determined for *Eucalyptus* leaf surfaces than the *δ*_*wax*_ calculated for *β*-diketones (approximately 16.6 MJ^1/2^ m^-3/2^). This may be attributed to the nano-structure of the *Eucalyptus* leaf surface, which decreases the *δ*_*θ*_ value in association with a high degree of surface roughness and hydrophobicity, as shown for various synthetic and natural materials [53].

### Solubility parameters of agrochemicals

The total solubility parameters (*δ*_*m*_) and solubility parameter components of the selected molecules are shown in Table 6. The water-soluble compounds urea and sorbitol have high *δ*_*m*_ values and major contributions from *δ*_*p*_ and especially *δ*_*h*_. A similarly high *δ*_*m*_ value was determined only for the non-systemic fungicide chlorothalonil, which also has the highest *δ*_*d*_ value of all the compounds considered.

**Table 6:** **Total solubility parameters (*δ***_***m***_**) and solubility parameter components of agrochemicals**

	**Solubility parameter components (MJ**^**1/2 **^**m**^**-3/2**^**)**			
Compound	*δ*_*d*_	*δ*_*p*_	*δ*_*h*_	*δ*_*m*_ (MJ^1/2^ m^-3/2^)
Urea	18.8	8.5	20.4	29.0
Sorbitol	17.3	6.1	31.3	36.3
Flutolanil	18.8	3.8	5.8	20.0
Fenoxycarb	19.1	4.0	6.1	20.4
Chlorothalonil	25.7	10.2	6.6	28.4
Formetanate	17.7	9.0	8.4	21.6
Esfenvalerate	18.8	2.6	6.2	20.0
*α*-Cypermethrin	22.6	4.1	6.5	23.9
Triton X-100	16.7	6.7	9.1	20.2
Brij 35	17.7	8.2	8.9	21.4
Genapol X-80	16.3	5.7	8.9	19.5

Insecticides (esfenvalerate, fenoxycarb, *α*-cypermethrin and formetanate) and the fungicide flutolanil have *δ*_*m*_ values ranging between 20 to 24 MJ^1/2^ m^-3/2^, with a major contribution from the *δ*_*d*_ component. In contrast, a higher *δ*_*m*_ was determined for the fungicide chlorothalonil. The results also indicate that compounds with different chemical structures such as flutolanil, fenoxycarb and esfenvarelate can have similar *δ*_*m*_ values.

The *δ*_*m*_ values of the three non-ionic surfactants are between 19.5 and 21.4 MJ^1/2^ m^-3/2^ and the differences among them are chiefly associated with the values of the *δ*_*d*_ and *δ*_*p*_ components. The major difference between Genapol X-80 and Brij 35 is related to *δ*_*p*_ (8.1 and 5.9 MJ^1/2^ m^-3/2^, respectively). The alkyl-phenol surfactant Triton X-100 lies between the values calculated for the two alkyl ethoxylates (*δ*_*m*_ = 20.2 MJ^1/2^ m^-3/2^).

### Affinity of agrochemicals for plant surfaces

**Figure 5:**
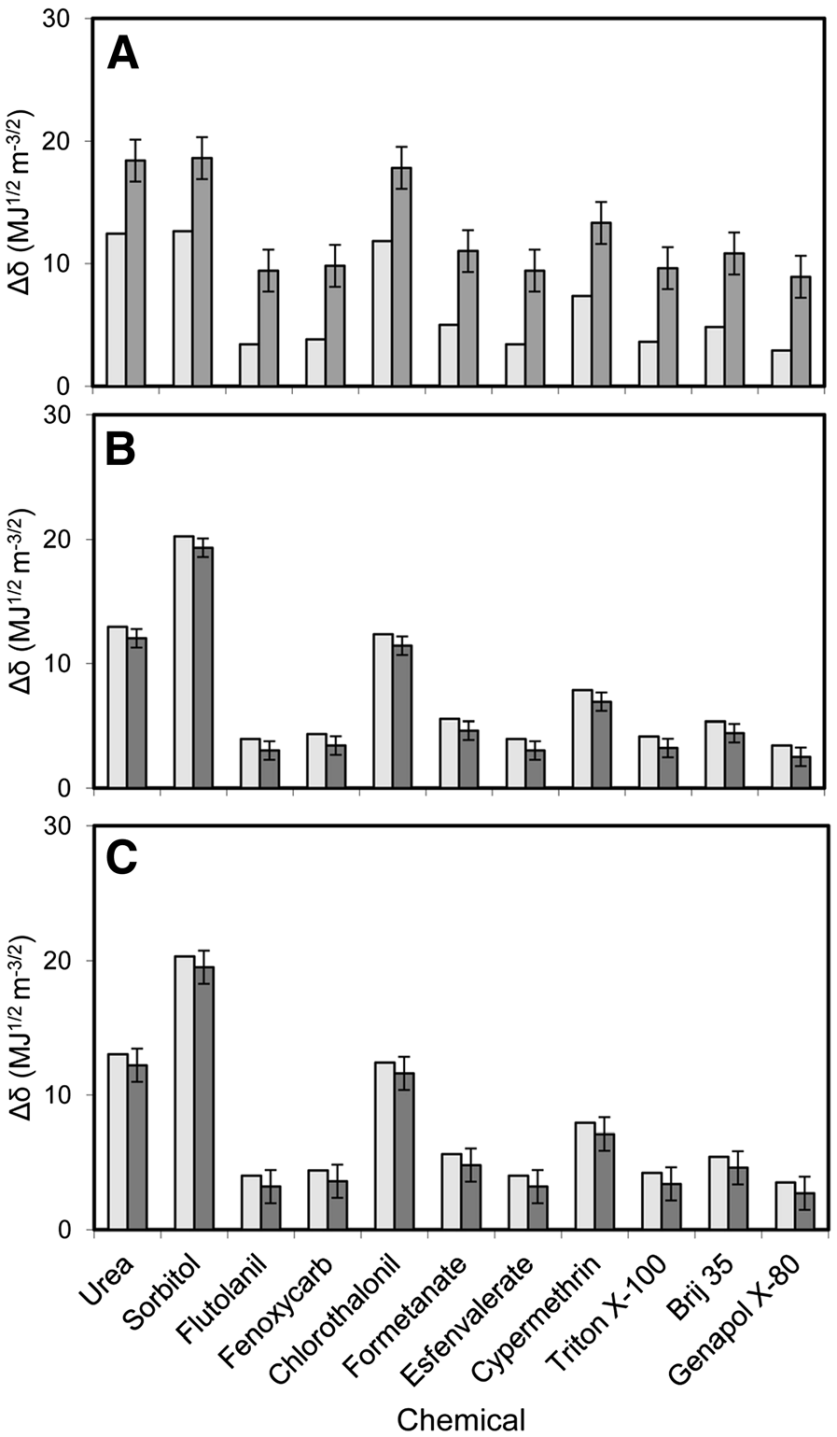
**Affinity of agrochemicals for *Eucalyptus *leaf (A), pepper (B) and peach fruit (C) surfaces.** Light grey bars represent the calculated *Δδ*_*wax*_, based on the solubility of agrochemicals in relation to the most abundant epicuticular wax compound (*δ*_*wax*_), and dark grey bars refer to *Δδ*_*θ*_(± standard errors)*,* calculated from contact angle measurements (*δ*_*θ*_).

Results concerning the affinity of agrochemicals for plant surfaces in relation to *δ*_*θ*_ and *δ*_*wax*_ are shown in Figure 5. The affinities of chemicals for the dominant epicuticular waxes present in *Eucalyptus* leaves, pepper and peach fruits are within a similar range (Figure 5A, B and C, light grey bars). The *n*-alkanes present in pepper and peach fruit surfaces provide a lower affinity for agrochemicals than the *β*-diketones of the *Eucalyptus* leaf.

The compounds with the lowest affinity for the epicuticular waxes covering the three plant materials analysed (i.e., those with the highest *Δδ*_*wax*_ values) are urea and sorbitol. Regarding the plant protection active ingredients, the highest affinity for epicuticular waxes was calculated for esfenvalerate and flutolanil, followed by fenoxycarb and formetanate. The compounds *α*-cypermethrin and chlorothalonil have higher *Δδ*_*wax*_ values and hence a lower affinity for the dominant waxes. Genapol X-80 is the surfactant with the highest affinity for epicuticular waxes, followed by Triton X-100.

The range of affinities of the agrochemicals for pepper and peach fruit surfaces based on contact angle measurements (*Δ δ*_*θ*_) is similar to the range predicted from the dominant epicuticular waxes (*Δδ*_*wax*_). In contrast, lower affinities of agrochemicals for the *Eucalyptus* leaf surface were estimated in relation to contact angle measurements, which take into account the combined effects of surface chemistry and roughness (Figure 5, dark grey bars).

### Solubility parameter gradient across the plant surface

The total solubility parameters (*δ*_*mn*_) and solubility parameter components of model cutin monomers and cell wall polysaccharides are shown in Table 7. The values estimated for free *ω*-hydroxy-fatty acids, D-glucose and D-galacturonic acid are higher than those calculated after monomer esterification or formation of glycosidic bonds, mainly because the H-bonding component is lower.

**Table 7:** **Total solubility parameters (*δ***_***mn***_**) and solubility parameter components of common cutin monomers and cell wall constituents**

	**Solubility parameter components (MJ**^**1/2 **^**m**^**-3/2**^**)**			
Compound	*δ*_*d*_	*δ*_*p*_	*δ*_*h*_	*δ*_*mn*_ (MJ^1/2^ m^-3/2^)
Solubility parameter range of cutin monomers (minimal and maximal values)				
16-Oxy, hexanodecanoate	15.9	2.2	5.9	17.2
9,10-Epoxy-18-oxy-octadecanoate	15.5	3.0	6.5	17.1
10,16-Oxy- hexanodecanoate^*^	15.7	3.3	7.8	17.8^*^
10-Oxy, 16-hydroxy-hexanodecanoate^**^	16.1	2.9	10.3	19.4^**^
9,12,18,-Oxy-octadecanoate^*^	15.6	4.1	7.1	17.7^*^
9-Oxy, 18,12-dihydroxy-octadecanoate^**^	16.3	3.8	10.9	20.0^**^
Solubility parameter range of cell wall polysaccharide monomers^***^				
D-Glucose	13.8	16.6	24.2	32.6
D-Polygalacturonic acid	15.0	14.7	23.3	31.3

^*^ minimal *δ*_*mn*_ assuming that all hydroxyl groups are esterified.

^**^ maximal *δ*_*mn*_ assuming only two ester bonds.

^***^assuming two glycosidic bonds.

The *δ*_*mn*_ values of the monomers were estimated by determining the possible minimum and maximum values in association with the potential occurrence of more than two ester bonds (e.g., on 9,10,18-tryhidroxy-octadecanoic acid). The presence of free hydroxyl groups will raise the value of *δ*_*h*_ and hence increase the total solubility parameter. This can be observed, for example, by contrasting 10,16-oxy-hexanodecanoate (three ester bonds) with 10-oxy, 16-hydroxy-hexanodecanoate (two ester bonds); the *δ*_*mn*_ values are 17.9 and 19.4 MJ^1/2^ m^-3/2^, respectively. Similarly, the monomer 9,12,18-oxy-octadecanoate (four ester bonds; 17.7 MJ^1/2^ m^-3/2^) has a lower *δ*_*mn*_ value than 9-oxy, 18,12-dihydroxy-octadecanoate (two ester bonds; 20.0 MJ^1/2^ m^-3/2^). The cutin monomers derived from 16-hydroxy-hexanodecanoic and 9,10-epoxy-18-hydroxy-octadecanoic acid can only form two ester bonds and have the lowest *δ*_*mn*_ parameter.

The total solubility parameters determined for polymerised D-glucose and D-galacturonic acid (around 32.2 MJ^1/2^ m^-3/2^) are remarkably above the range assessed for waxes and cutin, chiefly because of the major contributions of the *δ*_*p*_ and *δ*_*h*_ components. Methylation of the carboxylic group in the pectic compound leads to only a slight increase of the *δ*_*mn*_ parameter (data not shown).

**Figure 6:**
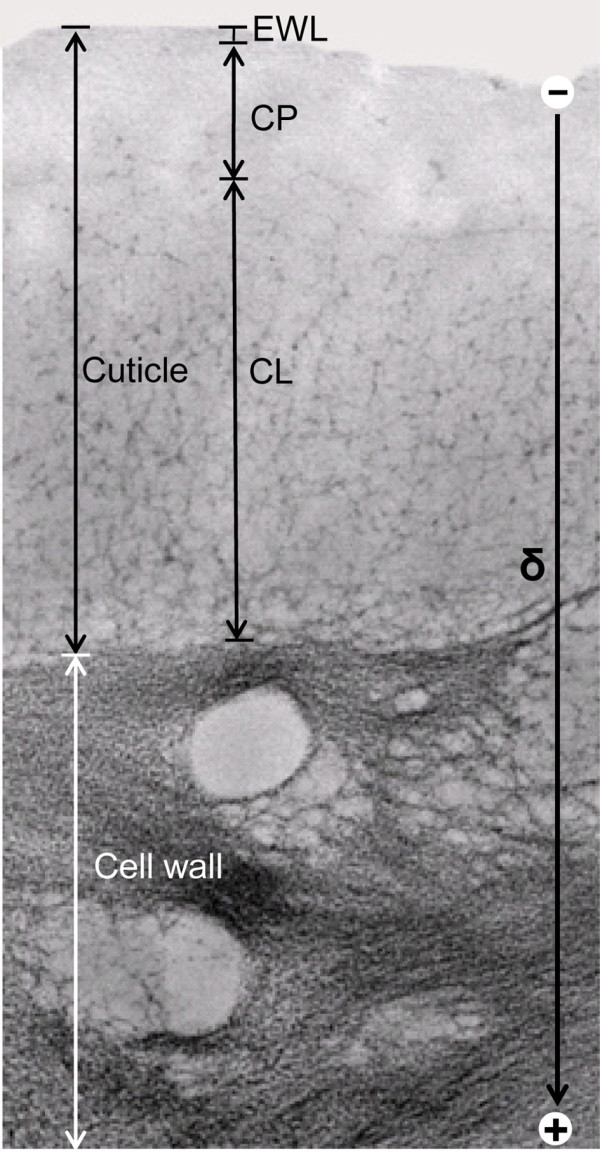
**Solubility parameter gradient model for the cuticle and cell wall covering the epidermis of plants.** The three cuticular layers are indicated as: EWL (epicuticular wax layer), CP (cuticle proper) and CL (cuticular layer). From the EWL (−) to the cell wall (+) there is a gradual solubility parameter increase. The TEM micrograph corresponds to a transverse section of a juvenile, adaxial *Eucalyptus globulus* leaf surface (×80,000).

By analysing the solubility parameter results estimated for the different materials covering epidermal cells and for the dominant epicuticular waxes present in the three species used for contact angle determinations, a solubility parameter gradient from the outer to the inner side of the cuticle can be expected as depicted in Figure 6. The epicuticular wax layer, which is in direct contact with the atmosphere, has the lowest solubility parameter value and may often lack polar and H-bonding components, as observed for *n*-alkanes. Cutin monomers usually form the cuticular matrix and their degree of polymerization can alter the total solubility parameter of the biopolymer (ranging between 17 and 20 MJ^1/2^ m^-3/2^). In the more external cuticular layers there are variable proportions of waxes and cutin (i.e., in the epicuticular wax layer and cuticle proper). Polysaccharides are present in the cuticular layer in direct contact with the cell wall. Therefore, according to current views on the composition of the materials (cuticle and cell wall) covering epidermal plant cells, a solubility parameter gradient is established from the external and more hydrophobic epicuticular wax layer towards the more hydrophilic internal cell wall.

## Discussion

In this study, a procedure for predicting the interactions among different structural plant surface constituents and agrochemicals, based on the estimation of solubility parameters, has been introduced for the first time in a plant science context. While prediction of solubility parameters is commonly used in membrane science [33] and also in pharmacology [36], this procedure has not so far been applied to estimating the surface properties of biological materials, with the exception of a few studies focused on human skin [37,38].

The permeability of a compound through a plant cuticle is the product of its solubility, which is a thermodynamic parameter reflecting the degree of interaction between that compound and the plant cuticle, and its diffusivity through the matrix of the plant cuticle, which is a kinetic parameter associated with the molecular size of the compound and the structure of the matrix. This study is only focused on analysing for the first time the solubilities (not the permeabilities) of model plant surfaces and chemical constituents in relation to agrochemicals of commercial significance, adopting a thermodynamic perspective.

Two different approaches have been followed to assess the solubility parameters of plant surfaces. One is based on contact angle measurements (*δ*_*θ*_), which reflect both physical and chemical effects associated with the topography and composition of the surface. The other is limited to considering the nature of the dominant epicuticular waxes covering the surface (*δ*_*wax*_). The three materials selected are good examples of the diversity of plant surface structures in relation to the variability of cell shapes and micro- and nano-structures on the cell surfaces. In addition, the similar chemical composition of the dominant waxes in pepper and peach fruits in contrast to the *β*-diketones prevailing in juvenile *Eucalyptus* leaves offers another interesting aspect to evaluate in the plant surfaces investigated. From an agrochemical viewpoint, substances with different activities, polarities, and degrees of complexity were assessed as model compounds.

### Plant surface properties

The adaxial *Eucalyptus* leaf surface is almost superhydrophobic and has a high degree of nano-roughness conferred by the dense network of wax nano-tubes [54]. However, the very hydrophobic surface of the highly pubescent peach variety analysed has a high degree of micro-roughness provided by the trichome network. In contrast, the pepper fruit surface has a smooth topography and it is more wettable than the other plant surface samples.

The determination of contact angles of three liquids enabled the three distinct plant materials to be compared by a novel approach [20]. Consequently, the three plant surfaces were characterised in terms of surface free energy, polarity, work of adhesion and solubility parameter. Fruit surfaces have the highest surface free energies and solubility parameter values, in contrast to the *Eucalyptus* leaf surface. However, the peach skin is the surface with the lowest polarity, which implies the lowest degree of potential polar and H-bonding interactions among the three surfaces analysed. The pepper surface has a significantly higher work of adhesion for water than the peach fruit and the *Eucalyptus* leaf surface. This indicates that water drops falling on the pepper fruit surface will be retained, in contrast to the repulsion of the drops falling on to peach fruit and especially *Eucalyptus* leaf surfaces. Therefore, the behaviour of the plant surfaces evaluated may bring some ecophysiological advantage to the plant organs and related species, which future studies should investigate further [20].

Furthermore, the proposed tools based on contact angle measurements of the three liquids may be useful for investigating plant surface dynamics during the growing season or as affected by plant biotic or abiotic stress factors. For example, epicuticular wax erosion in association with environmental pollution [29] or the deposition of aerosols and microorganisms [27,28] could increase the degree of heterogeneity and wettability of the surface, ultimately affecting plant-water relationships [55].

### Solubility parameters of cuticular components and agrochemicals

The solubility parameters of the dominant waxes in the analysed surfaces lie within the range 16.0 to 16.7 MJ^1/2^ m^-3/2^. Such a range is representative of a wide number of wax compounds such as *β*-diketones, *n*-alkanes, amyrins, and a few other additional compounds having aldehyde, ketone, and alcohol functional groups. The model cutin monomers evaluated have higher solubility parameters than waxes, varying between 19 and 22 MJ^1/2^ m^-3/2^ for the free *ω*-hydroxy-fatty-acids and possibly falling to 17 MJ^1/2^ m^-3/2^ after esterification of all functional hydroxyl groups. The model cellulose and pectin monomers have considerably higher solubility parameters than other cuticle constituents, which supports previous observations on the hydrophobicity of the cuticle as compared to the cell wall [2].

The presence of wax compounds such as *n*-alkanes renders the plant surface apolar. Despite the smooth topography and wettability of the pepper fruit, its *n*-alkane coating will only lead to the occurrence of dispersive interactions with surface-deposited materials and liquids, a phenomenon that can also be expected for peach fruits.

The different numbers of EO units in the surfactants evaluated had only a slight effect on the total solubility parameter. A correlation between surfactant solubility parameters and HLB or critical micelle concentrations has been reported [56,57]. The surfactant with the lowest solubility parameter (Genapol X-80) was recorded as having the lowest surface tension (approximately 27 mJ m^-2^ at 0.1%) while the highest surface tension (around 45 mJ m^-2^ at 0.1%) and solubility parameter were estimated for Brij 35.

It must be noted that water has a total solubility parameter of 47.9 MJ^1/2^ m^-3/2^[58] and that all chemicals except urea and sorbitol, which can be supplied at concentrations above 1%, were applied at approximately 0.1% concentrations. However, it can be expected that the chemicals assessed will interact with epicuticular waxes once sprayed on to plant surfaces as aqueous solutions, especially if surface-active agents are applied to improve contact between the solid and the liquid.

### Affinities of agrochemicals for plant surfaces

The affinities of agrochemicals for plant surfaces were evaluated on the basis of contact angle measurements and by considering the chemical structures of the dominant epicuticular waxes. According to Greenhalgh et al. [59], compounds with a solubility parameter difference (*Δδ*) below 7 MJ^1/2^ m^-3/2^ are likely to be miscible, while chemicals with a *Δδ* higher than 10 MJ^1/2^ m^-3/2^ are likely to be immiscible. The agrochemicals and plant surfaces selected in this study were found to have *Δδ* values ranging between 2.5 MJ^1/2^ m^-3/2^ (the highest affinity) and 26.6 MJ^1/2^ m^-3/2^ (no affinity). This indicates that such plant surfaces have a high affinity for some agrochemicals (Genapol X-80, flutolanil, esfenvalerate, Triton X-100, fenoxycarb, Brij 35, formetanate), which can readily penetrate into the plant organ (leaf or fruit in this case), and less affinity for other compounds (*α*-cypermethrin, sorbitol, urea and chlorothalonil).

The plant protective chemicals with the highest affinities for plant surfaces were found to be those with the lowest total solubility parameters. The high affinities of esfenvalerate, flutolanil and fenoxycarb for the surfaces evaluated make these compounds more prone to cuticular uptake and sorption into plant tissues. Since such plant protection products can be sprayed on to the leaves and fruits of agro-forest species, the estimation of solubility parameters could be used as a complementary tool for pesticide risk assessment. Therefore, the compounds with the lowest toxicity risk will be those with lower affinities for plant surfaces. The proposed methodology may be useful for improving the performance of foliar sprays of e.g., plant protection products, herbicides and fertilisers, taking into account their mode of action (e.g., systemic or contact) and the surface properties of the target organism (e.g., the plant, or surface pathogens and pests).

The surfactants selected in this study also have high affinities for plant surfaces, especially in the case of Genapol X-80. Surfactant solutions sprayed on to foliage have often been observed to be phytotoxic [60], which may be associated with the high solubility of some of these compounds in plant surfaces.

The lower affinities of agrochemicals for the *Eucalyptus* leaf estimated from contact angle measurements are due to the major roughness provided by the wax nano-tubes that densely cover the surface. These nano-tubes are also responsible for the high degree of hydrophobicity of the material [22,23]. In contrast, such differences between the affinities predicted from contact angle measurements and epicuticular wax chemistry were not observed for the peach and pepper fruit surfaces. The micro-scale roughness provided by the dense layer of trichomes covering the peach surface markedly increases the hydrophobicity of that surface, but seems to have limited effect on the solubility parameter.

### Solubility parameter gradient model

To gain insight into the characteristics of the cuticle and the cell wall by calculating solubility parameters, additional estimations were made for common cuticular matrix constituents and cell wall polysaccharides. To our knowledge, this is the first time in which the polar, dispersive and H-bonding properties of plant cuticular and cell wall constituents have been interpreted in quantitative terms. Owing to the properties of the dominant epicuticular waxes present in the three analysed plant materials, it is concluded that the solubility parameter increases with increasing depth from the epicuticular wax surface towards the cell wall. The solubility parameters determined for the model cellulose and pectin compounds are much higher than those for cutin monomers and epicuticular waxes but are still far away from the value of water.

Given the ubiquitous presence of waxes, cutin and polysaccharides in the layers covering plants’ epidermal cells [48], and assuming that the chemical constituents selected are representative of a wide range of species with hydrophobic surface properties and within the same range of potential alternative chemical components, a solubility parameter gradient is observed for a model plant surface, which can be applied to e.g., adaxial and abaxial leaf, fruit, flower or trichome surfaces that are covered with a cuticle. On the basis of thermodynamic principles, compounds with a low surface free energy (i.e., a low solubility parameter) will tend to migrate from the plant cell wall towards the epicuticular wax layer in order to decrease the Gibbs free energy [50]. This could be an alternative and/or complementary hypothesis to explain the migration of cuticular material (waxes and cutin) towards the air/plant interface, in contrast to cuticular transpiration as a driving force [13,14,17].

As noted by Scherbatskoy & Tyree [61], cuticular polymers contain polar and ionisable substituents, providing the cuticle with polar hydrophilic regions and ion exchange capacity. The topography and chemistry of epicuticular waxes generally provide a lower solubility parameter than the one prevailing in the cuticle proper and principally in the cuticular layer, where significant amounts of polysaccharides are present. The lack of polar and H-bonding functional groups in the dominant epicuticular waxes and a higher degree of monomer esterification in the cuticle matrix will tend to lower the total solubility parameter of the membrane by reducing the polar and H-bonding components. The presence of cutan can also decrease the solubility parameter of the cuticle matrix to some extent. According to Jeffree [9], cutanization (i.e., the gradual formation of cutan in the cuticle matrix) of the cuticular layer, as reported for the *Clivia minata* leaf cuticle [62], can arise from a maturation process involving the progressive modification of the previously deposited cutin and any embedded polysaccharides and waxes. The author suggests that the progressive reduction in reactivity of all components of the cuticular layer during cutanisation indicates that all types of polar functional groups are systematically eliminated during this maturation phase.

In order to calculate the solubility parameters, the properties of the trichomes covering the peach skin surface were considered. In this case the cuticular matrix is composed exclusively of cutin, in contrast to the high percentage of cutan in shaved cuticular membranes [20]. According to the cutan hypothesis proposed by Jeffree [9], the cuticular matrix of the peach fruit has a lower solubility parameter than cutin as the dominant cuticular matrix bio-polymer. However, the cuticular domain of the peach fruit cuticle will also have contributions from cutin and polysaccharides, which will gradually raise the total solubility parameter of the membrane as it comes closer to the cell wall. The cuticular matrix of pepper is mainly made of cutin [41], but an insoluble fraction likely to be cutan has also been identified [19]. No information is currently available on the composition of the *Eucalyptus globulus* leaf cuticle matrix, but the major reduction in solubility parameter associated with the nano-scale roughness of the *Eucalyptus* surface supports the occurrence of the solubility parameter gradient shown in Figure 6.

While most aerial plant surfaces are believed to be covered with a cuticle based on a cutin and/or cutan matrix, which contains variable amounts of waxes and polysaccharides, trials with more hydrophilic surfaces and different materials should be carried out in the future to estimate the solubility parameters of plant surface chemical constituents quantitatively.

## Conclusions

A novel method for predicting the interactions between plant surface structural constituents, plant surfaces and agrochemicals has been introduced, which was useful for predicting the solubilities of plant surface constituents and the affinities of agrochemicals for plant surfaces. Calculation of the solubility parameters of plant surface constituents led us to observe a solubility parameter gradient established from the cuticular surface towards the wall covering epidermal cells. Comparison of solubility parameters between cuticular and cell wall components will be helpful for clarifying the structure and development of the cuticle from an ontological viewpoint and also for establishing a relationship between the chemical composition and structure of the cuticular membrane, which is currently lacking. The methodology should also be of interest for multiple biological purposes and could help us understand surface phenomena on multiple biological materials.

## Abbreviations

EO: Ethylene oxide; HLB: Hydrophile-to-lipophile balance; SEM: Scanning Electron Microscopy; TEM: Transmission Electron Microscopy.

## Competing interests

The authors declare that they do not have any competing interests.

## Authors' contributions

MK had the preliminary idea of calculating solubility parameters for plant surfaces and agrochemicals both theoretically and in relation to contact angle measurements. VF selected the model chemicals, plant surfaces and plant surface constituents and carried out the experiments. Dynamic discussions between the two authors enabled solubility parameters and affinity between chemicals to be calculated and a solubility parameter model for the plant surface to be developed. Both authors contributed to writing and improving the paper and approved the final manuscript.

## References

[B1] Eichert T Fernández V Marschner P Uptake and release of mineral elements by leaves and other aerial plant parts Marschner’s Mineral Nutrition of Higher Plants 2012 San Diego: Academic Press 71 84

[B2] Javelle M Vernoud V Rogowsky PM Gwyneth CI Epidermis: the formation and functions of a fundamental plant tissue New Phytol 2011 189 17 39 10.1111/j.1469-8137.2010.03514.x 21054411

[B3] Burton RA Gidley MJ Fincher GB Heterogeneity in the chemistry, structure and function of plant cell walls Nat Chem Biol 2010 10 724 732 20852610 10.1038/nchembio.439

[B4] Domínguez E Heredia-Guerrero JA Heredia A The biophysical design of plant cuticles: an overview New Phytol 2011 189 938 949 10.1111/j.1469-8137.2010.03553.x 21374891

[B5] Fernández V Eichert T Uptake of hydrophilic solutes through plant leaves: current state of knowledge and perspectives of foliar fertilization Crit Rev Plant Sci 2009 28 36 68 10.1080/07352680902743069

[B6] Samuels AL Kunst L Jetter R Sealing plant surfaces: cuticular wax formation by epidermal cells Ann Rev Plant Biol 2008 59 683 707 10.1146/annurev.arplant.59.103006.093219 18251711

[B7] Pollard M Beisson F Li Y Ohlrogge JB Building lipid barriers: biosynthesis of cutin and suberin Trends Plant Sci 2008 13 236 246 10.1016/j.tplants.2008.03.003 18440267

[B8] Tyree MT Scherbatskoy T Tabor CA Leaf cuticles behave as asymmetric membranes. Evidence from the measurement of diffusion potentials Plant Physiol 1990 92 103 109 10.1104/pp.92.1.103 16667229 PMC1062254

[B9] Jeffree CH Riederer M, Müller C The fine structure of the plant cuticle Annual Plant Reviews, Volume 23. Biology of the Plant Cuticle 2006 Volume 23 Oxford: Blackwel 11 125

[B10] Kosma DK Bourdenx B Bernard A Parsons EP Lü S Joubès J Jenks MA The impact of water deficiency on leaf cuticle lipids of Arabidopsis Plant Physiol 2009 151 1918 1929 10.1104/pp.109.141911 19819982 PMC2785987

[B11] Yeats TH Buda GJ Wang Z Chehanovsky N Moyle LC Jetter R Schaffer AA Rose JKC The fruit cuticles of wild tomato species exhibit architectural and chemical diversity, providing a new model for studying the evolution of cuticle function Plant J 1012 69 655 666 22007785 10.1111/j.1365-313X.2011.04820.x PMC3736592

[B12] Jeffree CE Baker EA Holloway PJ Ultrastructure and recrystallization of plant epicuticular waxes New Phytol 1975 75 539 549 10.1111/j.1469-8137.1975.tb01417.x

[B13] Neinhuis C Koch K Barthlott W Movement and regeneration of epicuticular wax through plant cuticles Planta 2001 213 427 434 10.1007/s004250100530 11506366

[B14] Koch K Neinhuis C Ensikat HJ Barthlott W Self assembly of epicuticular waxes on living plant surfaces imaged by atomic force microscopy (AFM) J Exp Bot 2004 55 711 718 10.1093/jxb/erh077 14966216

[B15] Kolattukudy PE Biopolyester membranes of plants: cutin and suberin Science 1980 208 990 1000 10.1126/science.208.4447.990 17779010

[B16] Graça J Lamosa P Linear and branched poly(ω-hydroxyacid) esters in plant cutins J Agric Food Chem 2010 58 17 9666 9674 10.1021/jf1015297 20687563

[B17] Heredia-Guerrero JA Domínguez E Luna M Benítez JJ Heredia A Structural characterization of polyhydroxy fatty acid nanoparticles related to plant lipid biopolyesters Chem Phys Lipids 2010 163 3 329 333 10.1016/j.chemphyslip.2010.01.006 20123090

[B18] Villena JF Domínguez E Stewart D Heredia A Characterization and biosynthesis of non-degradable polymers in plant cuticles Planta 1999 208 181 187 10.1007/s004250050548 10333583

[B19] Johnson EJ Chefetz B Xing B Spectroscopic characterization of aliphatic moieties in four plant cuticles Comm Soil Sci Plant Anal 2007 38 2461 2478 10.1080/00103620701588841

[B20] Fernández V Khayet M Montero-Prado P Heredia-Guerrero JA Liakoloulos G Karabourniotis G Del Río V Domínguez E Tacchini I Nerín C Val J Heredia A New insights into the properties of pubescent surfaces: peach fruit as model Plant Physiol 2011 156 4 2098 2108 10.1104/pp.111.176305 21685175 PMC3149954

[B21] Wagner P Fürstner R Barthlott W Neinhuis C Quantitative assessment to the structural basis of water repellency in natural and technical surfaces J Exp Bot 2003 54 1295 1303 10.1093/jxb/erg127 12654881

[B22] Ensikat HJ Ditsche-Kuru P Neinhuis C Barthlott W Superhydrophobicity in perfection: the outstanding properties of the lotus leaf Beilstein J Nanotech 2011 2 152 161 10.3762/bjnano.2.19 PMC3148040 21977427

[B23] Bhushan B Jung YC Natural and biomimetic artificial surfaces for superhydrophobicity, self- cleaning, low adhesion, and drag reduction Progress Mater Sci 2010 56 1 108

[B24] Holloway PJ The effects of superficial wax on leaf wettability Ann Appl Biol 1969 63 145 153 10.1111/j.1744-7348.1969.tb05475.x

[B25] Pandey S Nagar PK Pattern of leaf surface wetness in some important medicinal and aromatic plants of western Himalaya Flora 2003 198 349 357 10.1078/0367-2530-00107

[B26] Aryal B Neuner G Leaf wettability decreases along an extreme altitudinal gradient Oecol 2010 162 1 9 10.1007/s00442-009-1437-3 19727830

[B27] Burkhardt J Hygroscopic particles on leaves: nutrients or desiccants? Ecol Monographs 2010 80 369 399 10.1890/09-1988.1

[B28] Burkhardt J Basi S Pariyar S Hunsche M Stomatal penetration by aqueous solutions – an update involving leaf surface particles New Phytol In press 10.1111/j.1469-8137.2012.04307.x 22985197

[B29] Kardel F Wuyts K Babanezhad M Wuytack T Adriaenssens S Samson R Tree leaf wettability as passive bio-indicator of urban habitat quality Environ Exp Bot 2012 75 277 285

[B30] Neinhuis C Barthlott W Seasonal changes of leaf surface contamination in beech, oak, and ginkgo in relation to leaf micromorphology and wettability New Phytol 1998 138 91 98 10.1046/j.1469-8137.1998.00882.x

[B31] Kerstiens G Cuticular water permeability and its physiological significance J Exp Bot 1996 47 1813 1832 10.1093/jxb/47.12.1813

[B32] Riederer M Friedmann A Riederer M, Müller C Transport of lipophilic non-electrolytes across the cuticle Annual Plant Reviews, Volume 23. Biology of the Plant Cuticle 2006 Oxford: Blackwell 250 279

[B33] Khayet M Suk DE Narbaitz RM Santerre JP Matsuura T Study on surface modification by surface-modifying macromolecules and its applications in membrane separation processes J Appl Polym Sci 2003 89 2902 2916 10.1002/app.12231

[B34] Khayet M Vazquez Alvarez M Khulbe KC Matsuura T Preferential surface segregation of homopolymer and copolymer blend films Surface Sci 2007 601 885 895 10.1016/j.susc.2006.11.024

[B35] Hansen CM 50 Years with solubility parameters — past and future Progress Organ Coatings 2004 51 77 84 10.1016/j.porgcoat.2004.05.004

[B36] Hancock BC York P Rowe RC The use of solubility parameters in pharmaceutical dosage form design Int J Pharma 1997 148 1 21 10.1016/S0378-5173(96)04828-4

[B37] Gröning R Braun FJ Three dimensional solubility parameters and their use in characterising the permeation of drugs through the skin Pharmazie 1996 51 337 341 8710956

[B38] Dias M Hadgraft J Lane ME Influence of membrane–solvent–solute interactions on solute permeation in skin Int J Pharma 2007 340 65 70 10.1016/j.ijpharm.2007.03.030 17467936

[B39] Bauer S Schulte E Thier HP Composition of the surface waxes from bell pepper and eggplant Eur Food Res Technol 2005 220 5 10 10.1007/s00217-004-1046-7

[B40] Kissinger M Tuvia-Alkalai S Shalom Y Fallik E Elkind Y Jenks MA Goodwin MS Characterization of physiological and biochemical factors associated with postharvest water loss in ripe pepper fruit during storage J Amer Soc Hort Sci 2005 130 735 741

[B41] Parsons EP Popopvsky S Lohrey GT Lü S Alkalai-Tuvia S Perzelan Y Paran I Fallik E Jenks MA Fruit cuticle lipid composition and fruit postharvest water-loss in an advanced backcross generation of pepper (*Capsicum* sp.) Physiol Plant 2012 146 1 15 25 10.1111/j.1399-3054.2012.01592.x 22309400

[B42] Li H Madden JL Potts BM Variation in leaf waxes of the Tasmanian *Eucalyptus* species - I. Subgenus Symphyomyrtus Biochem Systemat Ecol 1997 25 631 657 10.1016/S0305-1978(97)00044-6

[B43] Wirthensohn MG Sedgley M Jones GP Epicuticular wax of juvenile *Eucalyptus* leaves and headspace analysis of leaf volatiles J Essential Oil Res 2000 12 401 441

[B44] Jones TH Potts BM Vaillancourt RE Davies NW Genetic resistance of *Eucalyptus globulus* to autumn gum moth defoliation and the role of cuticular waxes Canad J Forest Res 2002 32 11 1961 1969 10.1139/x02-118

[B45] Rapley L Allen GR Potts BM Susceptibility of *Eucalyptus globulus* to *Mnesampela privata* defoliation in relation to a specific foliar wax compound Chemoecol 2004 14 3–4 157 163

[B46] Steinbauer MJ Davies NW Gaertner C Derridj S Epicuticular waxes and plant primary metabolites on the surfaces of juvenile *Eucalyptus globulus* and *E. nitens* (*Myrtaceae*) leaves Aust J Bot 2009 57 474 485 10.1071/BT09108

[B47] Kolattukudy PE Scheper T Polyesters in higher plants Advances in biochemical engineering/ biotechnology 2001 Berlin: Springer 4 49 10.1007/3-540-40021-4_1 11217409

[B48] Kolattukudy PE Cutin from plants. Biopolymers Online 2005 Weinheim: Wiley-VCH Verlag GmbH

[B49] O’Neill MA York WS Rose JKC The composition and structure of plant primary cell walls The Plant Cell Wall 2005 Oxford: Blackwell Publishing/CRC 1 54

[B50] Khayet M Chowdhury G Matsuura T Surface modification of polyvinylidene fluoride pervaporation membranes AIChE J 2002 48 2833 2843 10.1002/aic.690481211

[B51] van Krevelen DW te Nijenhuis K Cohesive properties and solubility Properties of Polymers: Their Correlation with Chemical Structure; Their Numerical Estimation and Prediction from Additive Group Contributions 2009 4 Oxford: Elsevier 189 227

[B52] van Krevelen DW Hoftyzer PJ Properties of polymers: their estimation and correlation with chemical structure 1976 2 Amsterdam: Elsevier

[B53] Nosonovsky M Bhushan B Superhydrophobic surfaces and emerging applications: non-adhesion, energy, green engineering Curr Opin Coll Interf Sci 2009 14 270 280 10.1016/j.cocis.2009.05.004

[B54] Wirthensohn MG Sedgley M Epicuticular wax structure and regeneration on developing juvenile *Eucalyptus* leaves Aust J Bot 1996 44 6 691 704 10.1071/BT9960691

[B55] Pariyar S Eichert T Goldbach HE Hunsche M Burkhardt J The exclusion of ambient aerosols changes the water relations of sunflower (*Helianthus annuus*) and bean (*Vicia faba*) plants Environ Exp Bot In press

[B56] Samaha MW Naggar VF Micellar properties of non-ionic surfactants in relation to their solubility parameters Int J Pharma 1998 42 1 9

[B57] Schott H Hydrophilic–lipophilic balance, solubility parameter, and oil–water partition coefficient as universal parameters of nonionic surfactants J Pharma Sci 1995 84 1215 1222 10.1002/jps.2600841014 8801337

[B58] Senichev VY Tereshatov VV Wypych G General principles governing dissolution of materials in solvents. 4.1 Simple solvent characteristics Handbook of Solvents 2001 Toronto: ChemTec 101 124

[B59] Greenhalgh DJ Williams AC Timmins P York P Solubility parameters as predictors of miscibility in solid dispersions J Pharma Sci 1999 88 1182 1190 10.1021/js9900856 10564068

[B60] Uhlig BA Wissemeier AH Reduction of non-ionic surfactant phytotoxicity by divalent cations Crop Prot 2000 19 13 19 10.1016/S0261-2194(99)00076-9

[B61] Scherbatskoy T Tyree MT Kinetics of exchange of ions between artificial precipitation and maple leaf surfaces New Phytol 1990 114 703 712 10.1111/j.1469-8137.1990.tb00442.x

[B62] Riederer M Schönherr J Development of plant cuticles fine structure and cutin composition of *Clivia miniata* Reg. leaves Planta 1988 174 127 138 10.1007/BF00394885 24221429

